# Hypercalcemia during renal replacement therapy after liver transplantation

**DOI:** 10.1186/cc9533

**Published:** 2011-03-11

**Authors:** J Matsumi, H Morimatsu, K Morita

**Affiliations:** 1 Okayama University Hospital, Okayama, Japan

## Introduction

Patients who suffer from acute kidney injury (AKI) show electrolyte abnormalities that can be corrected using renal replacement therapy (RRT). But some reports showed hypercalcemia during RRT and they reasoned this as the effect of citrate used for anticoagulant. We report eight post-liver transplantation (LT) recipients who suffered from AKI requiring RRT without citrate, but showed abnormal increase of ionized calcium (iCa) levels.

## Methods

We retrospectively identified the recipients who suffered from AKI requiring CRRT after LT. Then we picked up those who had increased iCa over 1.25 mmol/l as hypercalcemia (group H). We compared these recipients with those who matched in graft-recipient weight ratio (G/R) and intraoperative transfusion (units/kg) as controls (group N). Data were expressed as means with standard deviations. Analyses were made using Student's *t *test. We considered *P *< 0.05 statistically significant.

## Results

Among 250 recipients who had undergone LT in our hospital, 12 recipients received RRT. All RRT patients received nafamostat mesilate for anticoagulation. Eight patients had increased iCa (group H). All recipients in group H died during their index hospitalization. Compared with group N, group H had a higher iCa (1.3 ± 0.1 vs. 1.1 ± 0.0 mmol/l) and total bilirubin (T.Bil; 17 ± 9 vs. 4 ± 0 mg/dl). See Table 1.

**Table 1:** Characteristics

	Group H	Group N	*P *value
G/R	0.9 ± 0.4	0.9 ± 0.3	0.5
RCC	0.3 ± 0.3	0.3 ± 0.4	0.41
FFP	0.8 ± 0.7	0.6 ± 0.5	0.33
PLT	0.2 ± 0.3	0.3 ± 0.3	0.33
iCa	1.3 ± 0.1	1.1 ± 0.0	< 0.01
T.Bil	17 ± 9	4 ± 0	< 0.01

## Conclusions

We reported eight LT recipients who suffered from AKI and required RRT and had abnormally increased iCa levels without using citrate as anticoagulant. Only T.Bil was higher in the hypercalcemic group compared with the matched control. Because all of the eight hypercalcemic patients with CRRT died, this abnormality would be important for patient outcome.

